# Effects of salt stress on the rhizosphere soil microbial communities of *Suaeda salsa* (L.) *Pall*. in the Yellow River Delta

**DOI:** 10.1002/ece3.70315

**Published:** 2024-09-23

**Authors:** Yumiao Zhang, Huan Wang, Xinhan Zhang, Ziqi Feng, Junhua Liu, Yan Wang, Shuai Shang, Jikun Xu, Tao Liu, Longxiang Liu

**Affiliations:** ^1^ College of Biological and Environmental Engineering Shandong University of Aeronautics Binzhou China; ^2^ Binzhou Public Utilities Service Center Binzhou China; ^3^ Shandong Qianfa Agricultural Technology Co., Ltd. Binzhou China

**Keywords:** salt stress, soil microbiota, *Suaeda salsa*, Yellow River Delta

## Abstract

Studies have shown that the microbiome of saline‐tolerant plants plays a significant role in promoting salt stress in non‐saline‐tolerant plants, but the microorganisms are still unclear. In the present study, the microbial diversity changes in *Suaeda salsa* (L.) *Pall*. in the Yellow River Delta region were investigated. In the bacterial community, the dominant bacteria in the rhizosphere soil of the low‐saline soil (YDL), moderate‐saline soil (YDM), and high‐saline soil (YDH) groups were *Proteobacteria*, *Chloroflexi*, *Bacteroidota*, and *Actinobacteriota* (at the phylum level), while *Ascomycota* and *Basidiomycota* were the dominant fungi in the fungal community. At the family level, with the increase of salinity, the relative abundance of *Rhodobacteraceae* (bacterial community), *Thermoascaceae*, and *Phaffomycetaceae* (fungal community) gradually increased; and to the best of our knowledge, there are no reports on the relationship between *Thermoascaceae* and *Phaffomycetaceae* families with salt stress. At the genus level, *Salinimicrobium* (bacterial community) was the dominant bacterium in the rhizosphere soil of the YDL, YDM, and YDH groups, while with the increase of salinity, the relative abundance of *Byssochlamys* and *Wickerhamomyces* (fungal community) gradually increased, and to the best of our knowledge there are no reports on the relationship between *Byssochlamys* and salt stress. Salinity mainly affected the bacterial community abundance, but it had little effect on the fungi community abundance. The bacterial community of the YDH group was dominated by bacteria of unknown origin (52.76%), while bacteria of unknown origin accounted for 26.46% and 20.78% of the bacterial communities in the YDM and YDL groups, respectively. The fungi community of the YDH group was dominated by YDL group fungi (relative abundance of 44.44%), followed by YDM group fungi (29.42%) and fungi of unknown origin (26.14%). These results provide a better understanding of the rhizosphere microbial diversity of saline–alkali‐tolerant plants, laying a foundation for developing a saline–alkali‐tolerant plant microbiome.

## Abstract 2

Studies have shown that the microbiome of saline‐tolerant plants plays a significant role in promoting salt stress in non‐saline‐tolerant plants, but the microorganisms and their mechanisms are still unclear. In this study, the *Suaeda salsa* (L.) *Pall*. in Yellow River Delta region was selected to study the changes of microbial diversity. These results can provide valuable information for understanding the rhizosphere microbial diversity of saline–alkali‐tolerant plants, and lay a foundation for developing saline–alkali‐tolerant plant microbiome.

## 1 INTRODUCTION

Located in the temperate monsoon area, the Yellow River Delta is rich in animal and plant resources, and it plays an important role in maintaining biodiversity and ecological security of the lower Yellow River, Yellow Sea, and Bohai Sea (Shang, Hu, Liu, Zang, Chen, Gao, Li, Wang, Liu, Xu, & Zhang, 2022). The main characteristics of the Yellow River Delta are the high degree of land salinization, large saline–alkali land area, and wide distribution (Dong et al., 2023). The total land area of the Yellow River Delta is 2.15 million hectares, with 786,000 ha of cultivated land resources and 700,000 ha of undeveloped land resources. The undeveloped land is rich in natural resources, is a unique ecological environment, and has good climatic conditions, making it a valuable land resource in the east coast of China (Dong et al., 2023). The soil in this area is tidal soil formed by the alluvial Yellow River and the coastal salinized tidal soil. Due to the high salinization degree, many crops do not normally grow in this region. Therefore, development of saline–alkali land resources and comprehensive utilization of saline–alkali land can increase the cultivated land area, which is of great significance for ensuring food security.

As an extreme soil environment with high salinity and alkali, saline–alkali soil cannot provide a suitable living environment for animals and plants, but it provides a good habitat for extremophiles, such as saline‐tolerant or saline‐eating microorganisms (Li et al., 2023; Wang et al., 2023). Halophytes have unique saline–alkali tolerance mechanisms to cope with the effects of stress in the process of adapting to the saline–alkali environment. Among them, the rhizosphere microbiome, which is known as the second genome of plants, contributes to the growth and development of plants, and it also participates in plant immunity. Different plants recruit different microorganisms, and complex and dynamic interactions between microorganisms and plants under abiotic stress can promote crop growth and alleviate stress damage without causing harm to the environment (Wang et al., 2022). *Halomonas*, *Bacillus*, *Stenotrophomonas* sp., *Bacillus atrophaeus*, and *Planococcus soli* have been isolated from *Salicornia rubra*, *Sarcocornia utahensis*, *Allenrolfea occidentalis* (Kearl et al., 2019), *Carex distans* (Manh Tuong et al., 2022), Maize (Hou et al., 2022), and other plants; these microorganisms significantly improve the stress tolerance of *Alfalfa*, *Arabidopsis*, *MicroTom tomato*, and Maize under salt stress. Although these studies have suggested that halophyte rhizosphere microorganisms improve the salt tolerance of other heterologous crops, the mechanism of action remains unclear (Wang et al., 2022).

*Suaeda salsa* is an annual herbaceous plant belonging to the genus *Suaeda*, a pioneer plant in saline–alkali land. *S. salsa* is widely distributed in China, and its fleshy leaves and unique resistance mechanism make it highly salt‐tolerant and drought‐tolerant (Bai et al., 2010). *S. salsa* is an important wild plant resource. In the process of long‐term co‐evolution, the microorganisms from the high‐salt microenvironment in *S. salsa* may have mechanisms to alleviate the stress of adversity on plants. Bacteria closely related to plants grown under stress may have adapted to the stress and benefited the host plant (Chatterjee & Niinemets, 2022). Therefore, it is of great significance to investigate the resources of salt‐tolerant bacteria in this kind of environment. In the present study, the Illumina NovaSeq high‐throughput sequencing method was used to sequence 16S rRNA and internal transcribed spacer (ITS) genes of rhizosphere soil bacteria in saline–alkali soil in the Yellow River Delta region, and the differences between microbial community structure and samples were analyzed to explore the relationship between microbial community structure and salinity. Tax4Fun2 and FUNGuild (Fungi Functional Guild) were used to predict the function of microorganisms in the rhizosphere soil of *S. salsa*. The present findings provide support for bacterial and fungal growth in saline–alkali soil in the Yellow River Delta, as well as the application of biological improvement measures, laying a foundation for the research and development of soil amendments in the future. The present study provides a theoretical basis for the research of bacterial resources in extreme saline–alkali environment, and it has important significance for the exploration and exploitation of soil microbial resources in an extreme saline–alkali ecological environment.

## 2 MATERIALS AND METHODS

### 2.1 Sampling site

The soil samples were collected from the saline–alkali land of Kenli Street, Kenli County, Dongying City, Yellow River Delta (37.541716° N, 118.617623° E). This region is a warm temperate monsoon climate area with outstanding continental meteorological characteristics and significant differences among the four seasons. The average annual maximum temperature is 19.0°C, and the minimum temperature is 9.0°C. The soil type is mainly saline soil, and the distribution of the salt content in the land is uneven (between 0.3% and 1.5%). In addition, the vegetation is widely distributed, including *S. salsa* and the *Phragmites australis*.

### 2.2 Collection of samples and determination the EC and TDS values of soil

In August 2022, EC values was determined in sampling area, and low‐saline soil (YDL, 2–4 ds/m), moderate‐saline soil (YDM, 4–8 ds/m), and high‐saline soil (YDH, 8–16 ds/m) sampling areas were used to collect rhizosphere soil samples of *S. salsa* according to the report of Ivushkin et al. (2019) (Ivushkin et al., 2019; Li et al., 2015). In the sampling area, three or four 1 m × 1 m quadrats were selected, and five rhizosphere soil samples were collected from each quadrat. To take into account soil heterogeneity, five soil samples of *S. salsa* were mixed. One rhizosphere soil sample was obtained by mixing five rhizosphere soil samples from each quadrat. The rhizosphere soil sample was divided into two parts as follows: one part was used to determine the electrical conductivity (EC) and soil salt content (total dissolved solids, TDS); and the other part was placed in an ice box and immediately transferred to a laboratory freezer at −80°C.

The EC and TDS were measured by soil physicochemical indexes (Boudjabi & Chenchouni, 2022; Xiao et al., 2021; Zhao et al., 2022). The soil was air‐dried, milled, and sieved with a 2‐mm screen. The soil sample (W_1_ = 5 g) was then mixed with water at a ratio of 1:5 by full soaking and shaking. The EC and TDS were measured using mixed sample. The EC was established in the soil filtrate using a WTW/LF‐330 conductivity meter, which corrects the reading due to the temperature variation (Boudjabi & Chenchouni, 2022). For TDS measurement, the mixed sample was shaken and filtered all the liquid, dried at 105°C until the difference between the two weights was less than 0.0003 g, and weighed as W_2_. The TDS was calculated as W_2_/W_1_ (g/kg) (Xiao et al., 2021, 2023).

### 2.3 High‐throughput sequencing of the soil microbiome

The soil samples stored at −80°C were divided into 1.5‐mL Eppendorf tubes to set up the following replicates: three replicates for the YDL group (YDL1, YDL2, and YDL3), three replicates for the YDM group (YDM1, YDM2, and YDM3), and four replicates for the YDH group (YDH1, YDH2, YDH3, and YDH4). The V3 and V4 regions of the 16S rRNA gene were amplified by PCR using the following primers: 338F, ACTCCTACGGGAGGCAGCA; and 806R, GGACTACHVGGGTWTCTAAT. The following primers were to amplify the ITS region of fungi using PCR: ITS1F, CTTGGTCATTTAGAGGAAGTAA; and ITS2R, GCTGCGTTCTTCATCGATGC. Each reaction was performed in a total volume of 20 μL, which included 5–50 ng of DNA template, 0.3 μL of forward primer (10 μM), 0.3 μL of reverse primer (10 μM), 5 μL of KOD FX Neo Buffer, 2 μL of dNTPs (2 mM each), 0.2 μL KOD FX Neo, and ddH_2_O up to 20 μL. The thermocycler program was as follows: initial denaturation at 95°C for 5 min; 20 cycles of denaturation at 95°C for 30 s, annealing at 50°C for 30 s, and extension at 72°C for 40 s; and final step at 72°C for 7 min. The amplified products were purified with an Omega DNA purification kit (Omega Inc., Norcross, GA, USA) and quantified using an Qsep‐400 (BiOptic, Inc., New Taipei City, Taiwan, ROC). The amplicon library was paired‐end sequenced (2 × 250) on an Illumina NovaSeq 6000 (Beijing Biomarker Technologies Co., Ltd., Beijing, China).

### 2.4 Data analysis

The α diversity index was calculated using the picante (v1.8.2) package in R (v3.1.1) (Wang et al., 2021). One‐way analysis of variance (ANOVA) was used to determine differences in soil properties between different groups. The principal coordinates analysis (PCoA) diagram was drawn by QIIME software (1.8.0) with principal_coordinates.py scripts (Bolyen et al., 2019). The PERMANOVA analysis was calculated with the VEGAN (v2.5.3) package in R (v3.1.1) (Ni et al., 2021). Student's *t*‐test was used to calculate the differences in the α diversity and β diversity indices between the groups using SPSS (IBM SPSS Inc., Chicago, IL, USA) (Shang, Hu, Liu, Zang, Chen, Gao, Li, Wang, Liu, Xu, Zhang, Wu, & Tang, 2022). The Chao1 and Ace indices were used to measure species abundance, that is, number of species. The Shannon and Simpson indices were used to measure species diversity, which was influenced by species richness and community evenness in the sample community. The LDA effect size (LEfSe) was used to analyze the difference of bacterial and fungal community composition in different samples (Segata et al., 2011). In this study, python2.7.8 software and the scipy‐0.14.1 database were used to calculate the correlation between species and environmental factors by Pearson correlation coefficient. Significant difference was defined as *p* < .05 (Geng et al., 2022). The function of bacteria was annotated using Tax4Fun2 (v1.1.5) based on the KEGG database (Hu et al., 2020). FUNGuild (1.0) was used to annotate the fungal function (Li et al., 2020).

## 3 RESULTS

### 3.1 The EC and TDS values

**FIGURE 1:**
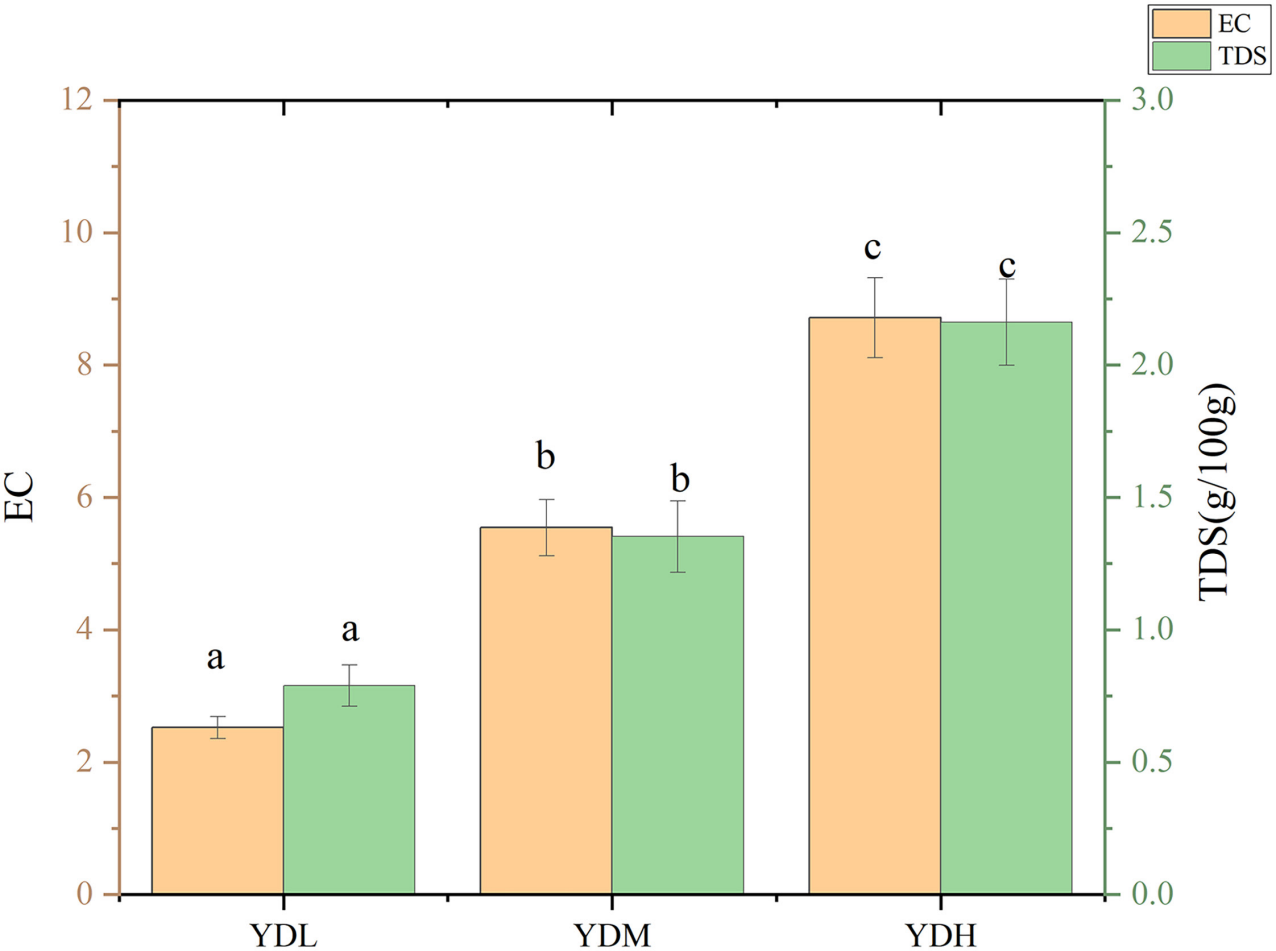
Differences in physical and chemical properties of soil under different treatments.

The EC and TDS values represent the soluble ion concentration of the sample and the total amount of various solid substances dissolved in water, respectively, which reflect the salt content of the soil. As shown in Figure 1, soil salt content in the YDL, YDM, and YDH groups gradually and significantly increased, which indicated that the salt concentration in the selected sampling sites had a certain gradient, suggesting that the test setting was reasonable.

### 3.2 Alpha diversity index

For the bacterial communities, 799,671 raw readings were obtained, and 795,092 clean reads were obtained after filtering the OTUs with low quality or counts less than 2. For fungal diversity, 712,464 raw readings were obtained, and 708,043 clean reads were filtered. The sequencing coverage of all samples was greater than 0.9997, indicating that the sequencing depth was adequate for describing the composition of fungal and bacterial communities. The diversity indices (Shannon and Simpson indices) and the computational richness indices (Chao1 and ACE indices) were used to calculate and analyze microbial α diversity. In the present study, the Simpson diversity index of bacteria and community diversity of fungi (Shannon, Simpson, Chao1 and ACE indices) were not significantly different among the YDL, YDM, and YDH groups (*p* > .05) (Table 1 and Table 2). The ACE, Chao1, and Shannon indices for bacteria were significantly higher in the YDL group compared to the YDM and YDH groups, and they were the lowest in the YDH group. Thus, the abundance of bacteria in the rhizosphere soil of *S. salsa* was lower in the high‐salinity group, and salinity had no significant effect on the abundance of fungi in the rhizosphere soil of *S. salsa*.

**TABLE 1:** Inter‐species α diversity index of bacterial.

Groups	ACE	Chao1	Simpson	Shannon
YDL	1330.31 ± 29.39	1329.07 ± 29.78	0.9974 ± 0.00029	9.4067 ± 0.0949
YDM	1299.45 ± 35.0361	1297.05 ± 34.59	0.9966 ± 0.00078	9.2475 ± 0.1351
YDH	1030.39 ± 75.19	1028.1 ± 74.53	0.9940 ± 0.00353	8.7388 ± 0.3185

**TABLE 2:** α diversity index between fungal species.

Groups	ACE	Chao1	Simpson	Shannon
YDL	442.33 ± 153.32	442.33 ± 153.32	0.9218 ± 0.059922	5.5854 ± 1.4734
YDM	590 ± 20.42	590 ± 20.42	0.9515 ± 0.034653	6.6190 ± 0.7845
YDH	610.75 ± 77.32	610.75 ± 77.32	0.9414 ± 0.049692	6.40493 ± 0.9414

### 3.3 OTU abundance

**FIGURE 2:**
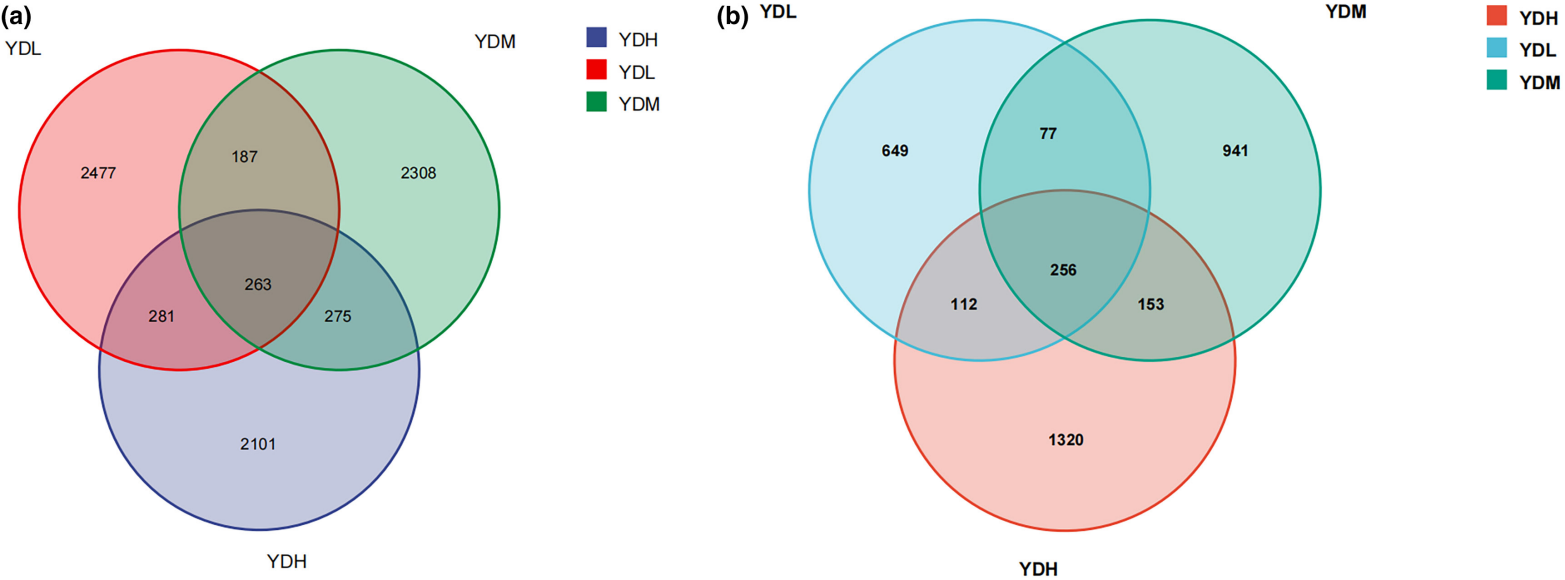
Common and unique OTU Venn diagrams of bacterial and fungal communities under different treatments (a: Bacterial community, b: Fungal community). Each circle represents sampled compartments. Values within intersections represent shared OTUs, values outside intersections represent unique OTUs.

For the bacterial community, 2920, 3033, and 3308 OTUs were identified in the YDH, YDM, and YDL groups, respectively (Figure 2a). For the fungal community, 1814, 1427, and 1094 OTUs were identified in the YDH, YDM, and YDL groups, respectively (Figure 2b). There were 263 common OTUs in the bacterial community, with 2101, 2308, and 2477 specific OTUs identified in the YDH, YDM, and YDL groups, respectively. There were 256 common OTUs in the fungal community, with 1320, 941, and 649 unique OTUs identified in YDH, YDM, and YDL groups, respectively. In the bacterial community, the highest number of OTUs was in the YDL group, while the highest number of OTUs for the fungal community was in the YDH group. These findings indicated that with the increase of salt concentration, the total number and unique number of bacteria decreased, while the total number and unique number of fungi increased.

### 3.4 Soil microbial community structure

**FIGURE 3:**
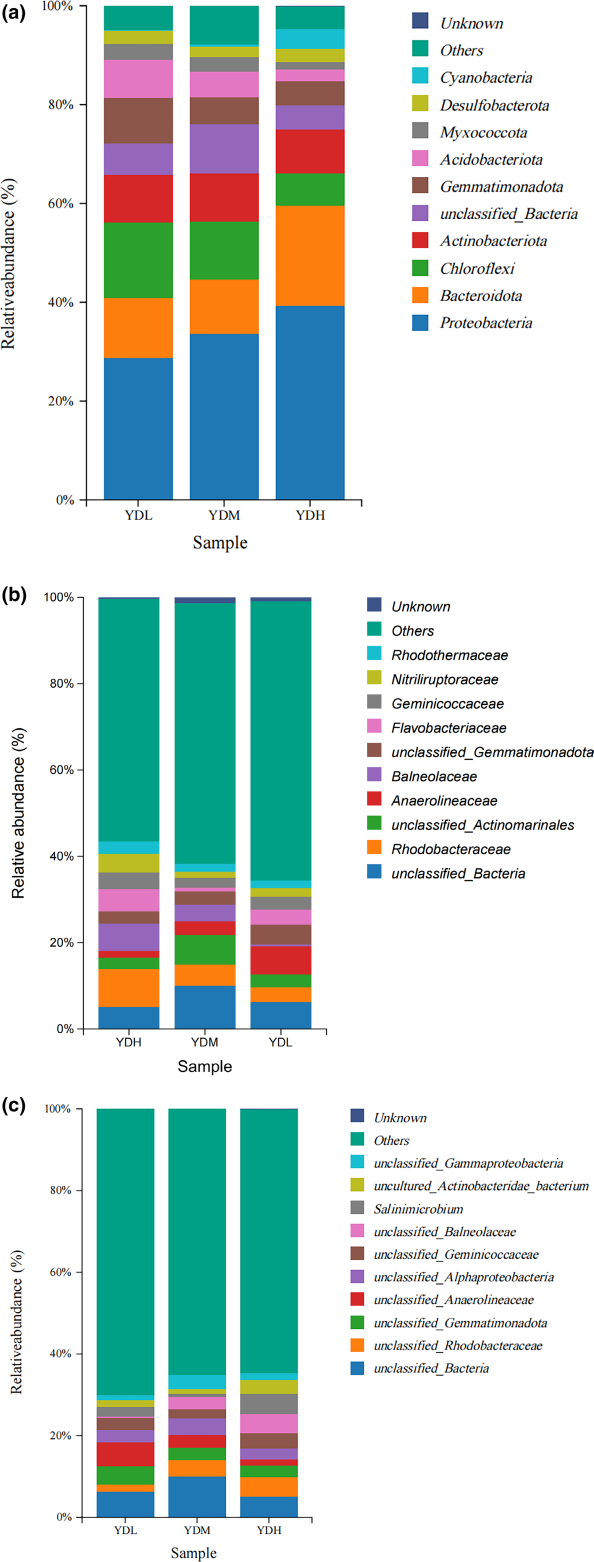
Relative abundance of the top 10 bacterial communities (a: At the phylum level, b: At the family level, c: At the genus level).

In the bacterial community, *Proteobacteria*, *Chloroflexi*, *Bacteroidota*, and *Actinobacteriota* were the dominant bacteria in the rhizosphere soil of the YDL, YDM, and YDH groups at the phylum level, with a total relative abundance of 65.9%, 66.0%, and 74.9%, respectively (Figure 3a). With the increase of salinity, the relative abundance of *Proteobacteria* gradually increased, while the relative abundance of *Chloroflexi* gradually decreased. These findings suggested that *Proteobacteria* may play an important role in the adaptation of *S. salsa* to high‐salinity soil. At the family level, except for the unclassified family and others, *Anaerolineaceae*, *Flavobacteriaceae*, and *Rhodobacteraceae* were the dominant bacterium in the rhizosphere soil of the YDL. *Rhodobacteraceae*, *Balneolaceae*, and *Anaerolineaceae* were the dominant bacterium in the rhizosphere soil of the YDM. *Rhodobacteraceae*, *Balneolaceae*, and *Flavobacteriaceae* were the dominant bacterium in the rhizosphere soil of the YDH. With the increase of salinity, the relative abundance of *Rhodobacteraceae* gradually increased, while the relative abundance of *Anaerolineaceae* gradually decreased. Thus, *Rhodobacteraceae* may be related to the adaptation of *S. salsa* to high‐salinity stress (Figure 3b). At the genus level, except for the unidentified genera, *Salinimicrobium* was the dominant bacterium in the rhizosphere soil of the YDL, YDM, and YDH groups, and the proportion of total bacteria abundance in YDH group was the highest. Thus, *Salinimicrobium* may be related to the adaptation of *S. salsa* to high‐salinity stress (Figure 3c).

**FIGURE 4:**
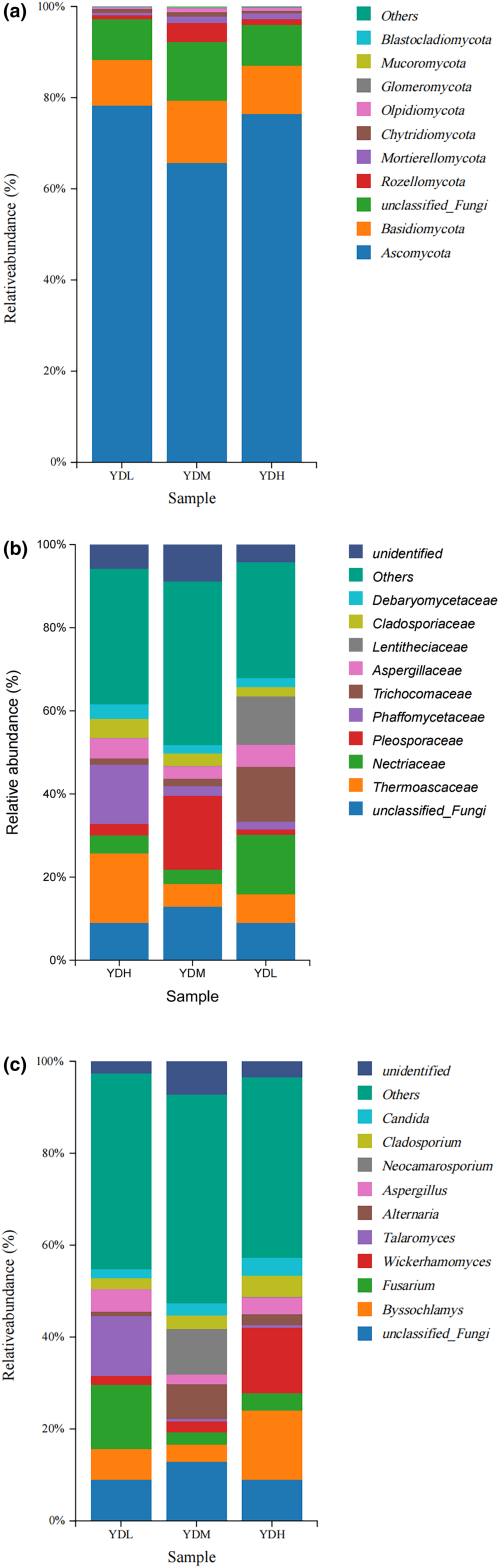
Relative abundance of the top 10 fungal communities (a: At the phylum level, b: At the family level, c: At the genus level).

In the fungal community, the dominant fungi in the rhizosphere soil of the YDL, YDM, and YDH groups were *Ascomycota* and *Basidiomycota* at the phylum level, with a total relative abundance of 88.3%, 79.4%, and 87.0%, respectively (Figure 4a). The total relative abundance of *Chytridiomycota* in the YDH group was lower than that in the YDL and YDM groups, while the total relative abundance of *Mortierellomycota* and *Olpidiomycota* in the YDM and YDH groups was higher than that in the YDL group. At the family level, except for the unclassified family and others, *Nectriaceae*, *Trichocomaceae*, and *Lentitheciaceae* were the dominant fungi in the rhizosphere soil of the YDL. *Pleosporaceae*, *Thermoascaceae*, and *Nectriaceae* were the dominant fungi in the rhizosphere soil of the YDM. *Thermoascaceae*, *Phaffomycetaceae*, and *Aspergillaceae* were the dominant fungi in the rhizosphere soil of the YDH. With the increase of salinity, the relative abundance of *Thermoascaceae* and *Phaffomycetaceae* gradually increased, while the relative abundance of *Nectriaceae* gradually decreased. Thus, *Thermoascaceae* and *Phaffomycetaceae* may be related to the adaptation of *S. salsa* to high‐salinity stress (Figure 4b). At the genus level, the dominant fungi in the YDL group were *Fusarium* and *Talaromyces*, with a total relative abundance of 27.1% (Figure 4c), which was significantly higher than that in YDM and YDH groups. The total relative abundance of dominant fungi was *Byssochlamys* and *Wickerhamomyces* in the YDH group (29.2%), which was significantly higher than that in YDL and YDM groups, indicating that *Byssochlamys* and *Wickerhamomyces* may be related to the adaptation to high‐salinity stress.

### 3.5 PCoA

**FIGURE 5:**
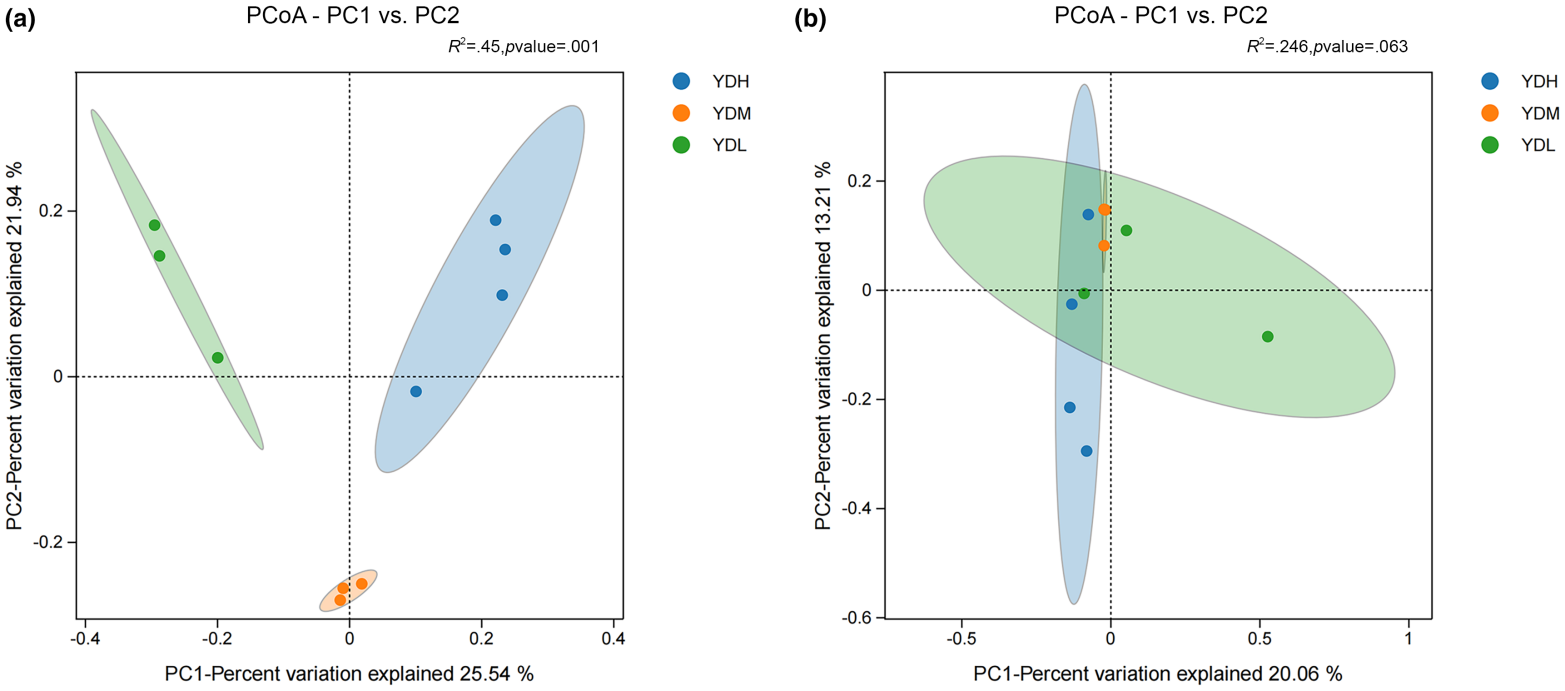
PCoA and PERMANOVA analysis based on the Bray–Curtis distance showing the variation in bacterial and fungal community structure (a: Bacterial community, b: Fungal community).

PCoA visually reflects the differences or similarities between different groups. In the present study, PCoA showed that the contribution rate of pc1 and pc2 to the bacterial community composition with different salinity was 25.54% and 21.49%, respectively (at the species level, Figure 5a). The bacterial communities in the YDM rhizosphere soil were closely related, indicating that their community composition was similar. In contrast, the rhizosphere soil bacterial communities of the YDL group were more dispersed, varied widely in community composition, and had lower community similarity. These results indicated that the distribution distance of the bacterial community in the rhizosphere soil of *S. salsa* varies under different salinity conditions, indicating that salinity influences the bacterial community in the rhizosphere soil of *S. salsa*. The contribution rate of pc1 and pc2 to the fungal community composition with different salinity was 20.06% and 13.21%, respectively (at the species level, Figure 5b). Under different salinity conditions, the relationship of fungal community composition in rhizosphere soil was the same as that of bacterial community. Among different replicates, the fungal community composition was most similar in the YDM group, while the difference was greatest in the YDL group. The results indicated that salinity influences the community composition of *S. salsa*.

PERMANOVA, also known as permutational MANOVA, is a statistical method used to analyze similarities between groups of multidimensional data. The *R* ^2^ obtained by PERMANOVA analysis represents the extent to which sample differences between different groups are explained and the ratio of group variance to total variance. In the present study, the *R* ^2^ value for the bacterial sample was 0.45 (*p* = .001) (at the species level, Figure 5a), and the *R* ^2^ value for the fungal sample was 0.246 (*p* = .063) (at the species level, Figure 5b). These findings indicated that the differences in salinity attributed to the differences of the bacteria in the rhizosphere soil, with significant differences among the YDL, YDM, and TDL groups, whereas the differences of the fungi in the rhizosphere soil among the three groups were not significantly attributed to the differences in salinity.

### 3.6 Correlation between bacterial species and soil physicochemical properties

**FIGURE 6:**
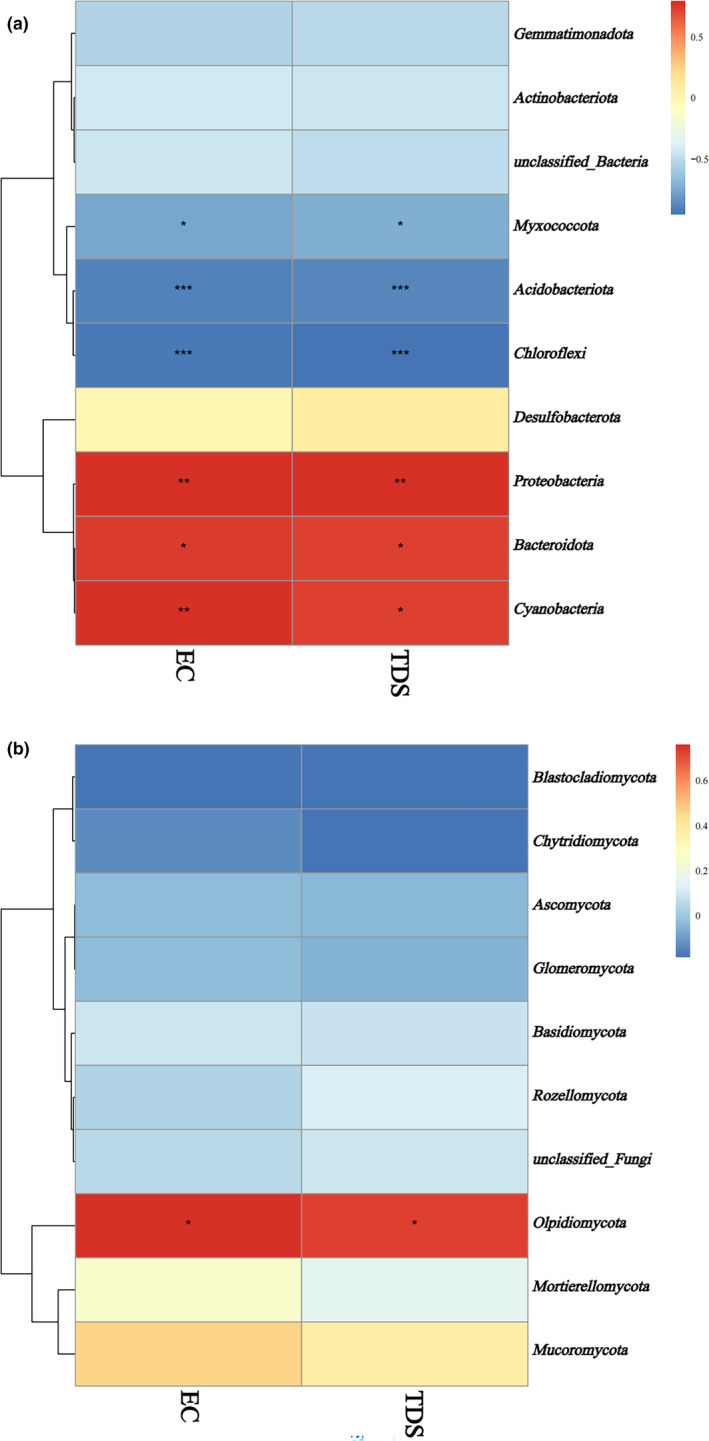
Relationship between the soil physicochemical properties and bacterial, fungal community (a: Bacterial community, b: Fungal community). * Means significant at *p* = .05 level, ** means significant at 0.01 level, *** means significant at 0.001 level.

Environmental factors have certain effects on soil microorganisms. The relative abundance of bacteria, relative abundance of fungi, EC value, and TDS were investigated to determine the correlation of bacterial and fungal species with soil physicochemical properties. The results showed that *Myxococcota*, *Acidobacteriota*, *Chloroflexi*, *Proteobacteria*, *Bacteroidota*, and *Cyanobacteria* were significantly correlated with the EC value and TDS in the bacterial community (Figure 6a), while the *Olpidiomycota* species was associated with the EC value and TDS in the fungal community (Figure 6b), and the total relative abundance of *Olpidiomycota* in the YDM and YDH groups was higher than that in the YDL group.

### 3.7 LEfSe analysis

**FIGURE 7:**
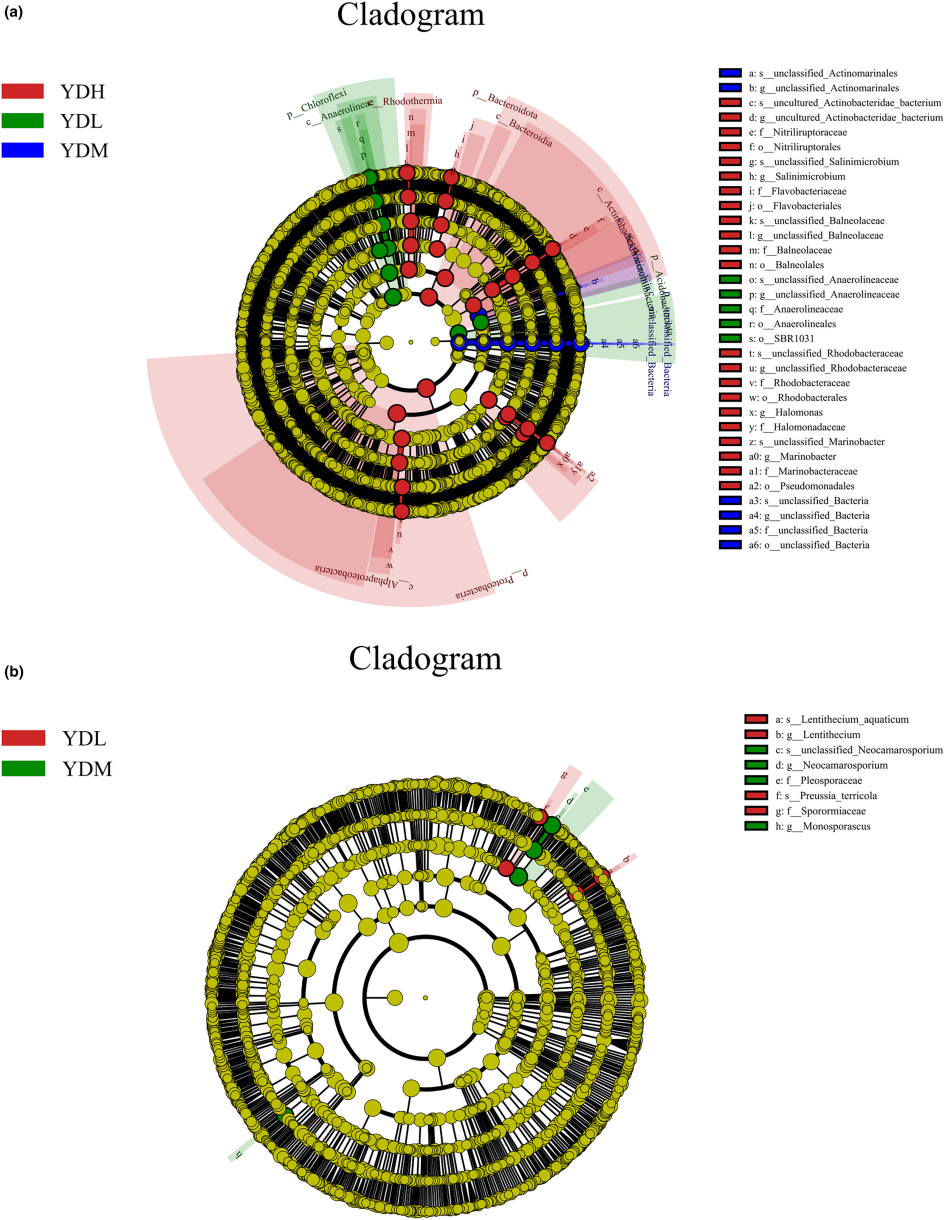
LEfSe analysis at different taxonomic levels among different groups (a: Bacterial community, b: Fungal community). Different colored dots represent the taxa with significant differences among different samples.

LEfSe analysis can identify biomarkers that have statistical differences between different groups. Bacterial community sequencing showed that *Acidobacteriota*, *Chloroflexi* (at the phylum level), and unclassified_*Anaerolineaceae* (at the genus level) played an important role in the YDL group (Figure 7a). In addition, *Actinobacteriota* (at the phylum level) and unclassified_*Actinomarinales* (at the genus level) played an important role in the YDM group, whereas *Proteobacteria*, *Bacteroidota*, *Actinobacteriota* (at the phylum level), unclassified_*Balneolaceae*, uncultured_*Actinobacteridae*, *Salinimicrobium*, unclassified_*Rhodobacteraceae*, and *Marinobacter* (at the genus level) played an important role in the YDH group. Fungal community sequencing (Figure 7b) showed that *Ascomycota* (at the phylum level), *Preussia*, and *Lentithecium* (at the genus level) played an important role in the YDL group, whereas *Ascomycota* (at the phylum level), *Neocamarosporium*, and *Monosporascus* (at the genus level) played an important role in the YDM group.

### 3.8 Prediction of bacterial and fungal community function in the rhizosphere of *S. salsa*

**FIGURE 8:**
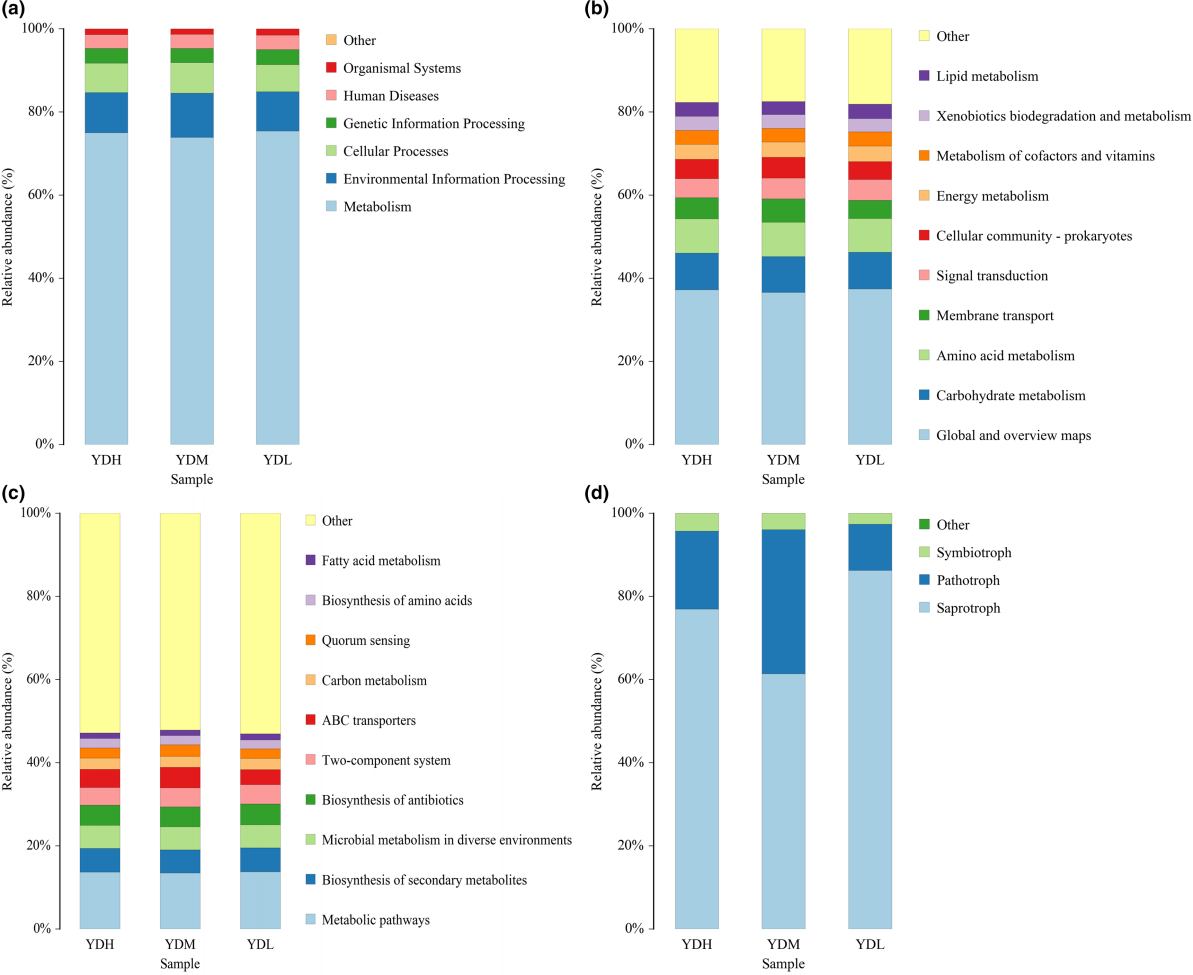
Classification of the function of rhizosphere bacteria and fungi in *Suaeda salsa*. (a) The primary metabolic pathway functional genes of the rhizosphere bacteria; (b) The bacterial functional annotations of the secondary metabolic pathways; (c) The bacterial functional annotations of the tertiary functional layer; (d) The function of fungi.

Tax4Fun was used to annotate the function of bacteria based on the KEGG database (Figure 8), and six types of primary metabolic pathway functional genes were obtained, including metabolism, environmental information processing, cellular processes, human diseases, genetic information processing, and biological systems. Under the three salinity conditions, the primary metabolic pathway functional genes of the rhizosphere bacteria were related to metabolism, with an average relative abundance of 73.88%–75.46%, followed by environmental information processing, with an average relative abundance of 9.38%–10.64% (Figure 8a). The bacterial functional annotations of the secondary metabolic pathways (relative abundance >5%) were as follows: global and overview maps of carbohydrate metabolism; amino acid metabolism; membrane transport and signal; and global and overview maps of transduction signal transduction (Figure 8b). In the tertiary functional layer, the rhizosphere bacterial community obtained a total of 314 metabolic pathways, which mainly involved metabolic pathways (13.51%–13.75%), biosynthesis of secondary metabolites (5.56%–5.74%), and microbial metabolism in diverse environments (5.55%–5.58%) (Figure 8c). There was no significant change trend in biological metabolic pathways under salt stress.

FUNGuild was used to annotate the function of fungi (Figure 8d), which were divided into saprotroph, pathotroph, and symbiotroph fungi according to the trophic mode of fungi. The rhizosphere of the three different salinities was dominated by saprotrophic fungi (with an average relative abundance of 61.38%–86.23%), followed by pathotrophic fungi (with an average relative abundance of 11.13%–34.69%) and symbiotic fungi (with an average relative abundance of 2.64%–4.28%). Among the three salinity groups, the abundance of fungi varied greatly among the three nutritional modes.

## 4 DISCUSSION

### 4.1 Soil microbial community structure of *S. salsa* in coastal saline–alkali land

Similar to the human gut microbiome, rhizosphere microbes are known as the second or extended microbiome genome of the plant host. Rhizosphere microorganisms play an important role. Rhizosphere microorganisms can promote the absorption and transport of nutrients from soil, as well as increase plant immunity and improve plant tolerance to biological and abiotic stresses. As a plant endemic to saline–alkali land, *S. salsa* tolerates 25 g/kg salinity, allowing it to grow in coastal beaches (Jia et al., 2021). Rhizosphere microorganisms play a key role in the tolerance to salt stress. After isolating various microorganisms from *S. salsa*, researchers have inoculated these microorganisms into maize and other plants, resulting in significant improvement in the stress tolerance of maize (Krishnamoorthy et al., 2016; Xu et al., 2022). Exploration of the rhizosphere microbial composition and analysis of rhizosphere microorganisms that play a key role in saline–alkali soil salt stress tolerance of *S. salsa* will aid the development of saline–alkali soil agriculture.

In the present study, high‐throughput sequencing technology was used to investigate the microbial diversity in saline–alkali rhizosphere soil in the Yellow River Delta under different salinity conditions. The rhizosphere fungal community was mainly composed of *Ascomycota* and *Basidiomycota* under different salinity conditions. Consistently, Wang and Guo (2016) and Liu, Wu, et al. (2023) reported that *Ascomycetes* and *Basidiomycota* are the dominant fungal group in the saline–alkali soil of the Yellow River Delta (Liu, Wu, et al., 2023; Wang & Guo, 2016), and continuous planting of *S. salsa* significantly increased the relative abundance of *Ascomycota* (Zhao et al., 2024). *Ascomycetes* exist widely in nature and have rich and diverse functions, and *Ascomycetes* are the most abundant flora in most saline–alkali soil samples (Wu et al., 2018). Among soil fungi, *Ascomycota* is the dominant phylum, and most of the species are saprophytic microorganisms, which play an important role in degrading soil organic matter. *Ascomycota* was the most dominant fungal phylum in saline–alkali soil samples in coastal areas, while *Ascomycota* and *Basidiomycota* were the most dominant fungal phyla in inland areas. Thus, *Ascomycetes* can adapt to survive in various saline environments, and *Basidiomycetes* predominate in a few saline soils (Si et al., 2011). At the family level, with the increase of salinity, the relative abundance of *Thermoascaceae* and *Phaffomycetaceae* gradually increased, while the relative abundance of *Nectriaceae* gradually decreased. Thus, *Thermoascaceae* and *Phaffomycetaceae* may be related to the adaptation of *S. salsa* to high‐salinity stress. At present, to the best of our knowledge there are no reports on the relationship between these two families and salt stress. At the genus level, with the increase of salinity, the relative abundance of *Byssochlamys* and *Wickerhamomyces* gradually increased, indicating that *Byssochlamys* and *Wickerhamomyces* may be related to the adaptation to high‐salinity stress. And *Wickerhamomyces* genus was found in spontaneous fermentation in 10% salt brine (Parafati et al., 2022; Penland et al., 2022), but, to the best of our knowledge there are no reports on the relationship between *Byssochlamys* and salt stress.

It has been reported that bacterial communities in saline–alkali soil samples mainly belong to Proteobacteria, Actinobacteria, Firmicutes, Bacteroidetes, Cyanobacteria, Blastomonas, Gemmatimonadetes, Acidobacteria, Thermomicrobia, Planctomycetes, and Chloroflexi (Yu et al., 2019). In the present study, the bacterial community was mainly composed of Proteobacteria, Chloroflexi, Bacteroidota, and Actinobacteriota. Furthermore, Proteobacteria, Bacteroidota, and Acidobacteriota were significantly correlated with the EC value and TDS in the bacterial community. This is consistent with the report of Liu et al. (2020), which accounts for 75.3% of the total bacterial community content in the intertidal zone in Yellow River Delta (Liu et al., 2020; Liu, Wu, et al., 2023). With the increase of salinity, the total relative abundance of Proteobacteria gradually increased, which agreed with previous bacterial diversity studies, showing that Proteobacteria had the highest abundance (>38%) in the Yellow River Delta, the Yangtze River Delta, and other wetlands, and is positive related to soil salinity (Chi et al., 2021; Liu, Zeng, et al., 2023; Yang et al., 2021). Actinobacteriota, Proteobacteria, and Bacteroidota are typical copiotrophs bacteria, while Acidobacteria and Chloroflexi are typical oligotrophic bacteria (Liao et al., 2023; Stone et al., 2023). At the family level, with the increase of salinity, the relative abundance of Rhodobacteraceae gradually increased, while the relative abundance of Anaerolineaceae gradually decreased. This is consistent with the report of Song et al. (2022), the family Rhodobacteraceae was abundant in low‐saline salterns (45–80 g/L) (Song et al., 2022). Thus, Rhodobacteraceae may be related to the adaptation of *S. salsa* to high‐salinity stress. At the genus level, Salinimicrobium had the highest relative abundance among bacterial communities under different concentrations of salt stress, which agreed with a previous study, reporting that Salinimicrobium is abundant in saline–alkali soil (Zhang et al., 2024). Salinimicrobium plays an important role in promoting plant growth, and it can change the growth environment of plants by releasing various beneficial elements (Zhang et al., 2023).

### 4.2 Effects of environmental factors on soil microbial community structure and microbial function

Vegetation type, soil physical properties, soil chemical properties, latitude gradient, soil temperature, or climate change can affect soil microbial community structure (Fang et al., 2017; Wu et al., 2019; Zhang et al., 2017). In the present study, correlation heatmap analysis was used to analyze if TDS and EC explained variation in relative abundance of fungal or bacterial groups. EC and TDS caused the differences of soil bacterial community structure in this study. PCoA showed that salinity attributed to the different composition of microbial community structure between samples, and the results were consistent with *Phragmites communis* (Zhao et al., 2020) and *Glycyrrhiza uralensis* (Du et al., 2016), two typical halophytes. Consistent with the heatmap analysis, LEfSe analysis indicated that the bacterial communities of the YDL and YDM groups showed a significant positive correlation with EC, which indicated that *Acidobacteriota*, *Chloroflexi*, and *Actinobacteriota* can survive in low‐ and medium‐salinity soil. Thus, the saline–alkali environment provides a living environment for these bacteria. Therefore, understanding the adaptive environment of microorganisms is helpful to identify and utilize microbial resources (Booker et al., 2023; Liu, Wu, et al., 2023; Liu, Zeng, et al., 2023;Ye et al., 2009).

The diversity and richness indices showed that the high‐salinity group had low bacterial abundance in the rhizosphere soil but that salinity had no significant effect on the fungal abundance. Moreover, the number of OTUs increased with the salt concentration. With the increase of salinity, the total number and unique number of bacteria decreased, whereas the total number and unique number of fungi increased. PCoA showed that, compared to other salinity groups, the microbial community composition of different biological replicates under moderate salt concentration was more similar. And the present study predicted the function of rhizosphere microorganisms in *S. salsa* under different salinity conditions. Among them, saprophytic fungi accounted for the largest proportion. In addition, the functional composition of rhizosphere bacteria was relatively stable under different salinity conditions, while rhizosphere fungi were significantly affected by salinity. These results indicated that although there were some differences in the composition of bacteria among the different groups, bacteria with similar functional prediction were obtained from the *S. salsa* rhizosphere soil. This may be because bacterial communities are in some cases not resistant to disturbances, and the functional redundancy among bacterial taxa can result in a situation where composition changes in response to the disturbance whereas functioning remains stable (Berga et al., 2017).

## 5 CONCLUSIONS

The present study sampled soil under different salinity conditions in the Yellow River Delta to analyze the community composition, soil microbial community functional characteristics, and the correlation of the microbial community with soil EC and TDS. Salinity affected the abundance of the bacterial community but had no significant effect on the abundance of the fungal community. With higher salinity, the total and unique number of bacteria significantly decreased, while the total and unique number of fungi increased. For the bacterial community, the *Rhodobacteraceae* (at the family level) *and Salinimicrobium* (at the genus level) may be related to the adaptation of salt stress in *S. salsa*. For the fungal community, *Thermoascaceae*, *Phaffomycetaceae* (at the family level), *Byssochlamys*, and *Wickerhamomyces* (at the genus level) may be related to the adaptation of salt stress in *S. salsa*. And to the best of our knowledge, there are no reports on the relationship between *Thermoascaceae*, *Phaffomycetaceae* families and *Byssochlamys* (fungal community) with salt stress. This provides possible new genera or families for the screening of saline–alkali‐tolerant growth‐promoting microorganisms. Besides, there were some differences in the composition of microorganisms among the different samples, but microorganisms with similar functions were recruited under salt stress. These results provide a better understanding of the rhizosphere microbial diversity of saline–alkali‐tolerant plants, laying a foundation for developing a saline–alkali‐tolerant plant microbiome.

## AUTHOR CONTRIBUTIONS

**Yumiao Zhang:** Conceptualization (equal); methodology (equal). **Huan Wang:** Investigation (equal). **Ziqi Feng:** Data curation (equal). **Xinhan Zhang:** Writing – original draft (equal). **Junhua Liu:** Investigation (equal). **Yan Wang:** Data curation (equal). **Shuai Shang:** Writing – original draft (equal). **Jikun Xu:** Writing – review and editing (equal). **Tao Liu:** Project administration (equal). **Longxiang Liu:** Conceptualization (equal); funding acquisition (equal); methodology (equal).

## CONFLICT OF INTEREST STATEMENT

The authors declare no conflicts of interest.

## Data Availability

All sequences analyzed in the present study can be assessed in the SRA database (https://www.ncbi.nlm.nih.gov/sra) under the accession number SRR27792911–SRR27792920 and SRR27793005–SRR27793014.
